# Comparison of baseline drifts using three reflector blocks versus using a single reflector block for the calibration of wall‐mounted Respiratory Gating for Scanner (RGSC) camera integrated with a CT

**DOI:** 10.1002/acm2.14199

**Published:** 2023-11-14

**Authors:** Bei Liu, Chengyu Shi, Maneesha Prakash, Bryan Gonzalez, Ari Kassardjian, Ji Kim, Paul Mandelin, Terence Williams, An Liu

**Affiliations:** ^1^ Division of Radiation Oncology City of Hope National Medical Center Duarte California USA

**Keywords:** baseline drift, calibration, RGSC, wall‐mounted camera

## Abstract

**Background:**

The calibration of the Respiratory Gating for SCanner (RGSC) system is critical to achieve better and more stable accuracy. The current procedure for a wall‐mounted RGSC system has a relatively large residual error.

**Purpose:**

To compare the baseline drifts in the image acquisition of DIBH using three reflector blocks versus using a single reflector block in the calibration of a wall‐mounted RGSC camera system.

**Materials and methods:**

Varian provides a calibration plate with three rows of calibration points: each row is separated by 15 cm longitudinally and by 10 cm laterally. In Varian's single‐block calibration method, the reflector block was first placed on the center point of the calibration plate and aligned with the scanner isocenter. The calibration took a picture of the block, then placed the block on the other eight points sequentially. In the proposed three‐block method, we placed three reflector blocks on the center row, with the center block aligned with the isocenter, and we took a picture of the center block by manually blocking the other two blocks in calibration. By moving the couch longitudinally in or out 15 cm, the calibration goes through all nine points. Monte Carlo simulation was done using Matlab to analyze the calibration matrix eigenvalue characteristics.

**Results:**

For a typical scan length of 40 cm of DIBH, the residual baseline drift in simulated DIBH is 0.02 ± 0.03  versus 0.30 ± 0.12 cm for three‐block calibration and single‐block calibration, respectively. To achieve 0.5 mm tolerance for the eigenvalue, the laser and reflector box should be within ±3 mm uncertainties based on the eigenvalue simulation.

**Conclusion:**

Three‐block calibration method effectively removes baseline drift caused by couch movement in DIBH/4D CT scan for the wall‐mounted camera while the single‐block calibration method still has significant residual baseline drift.

## 1 INTRODUCTION

Respiratory motion management techniques such as 4DCT, gating, and deep inspiration breath hold (DIBH) have been widely used in external beam radiation therapy for lung cancer,^1^, ^2^ liver cancer,^3^, ^4^, ^5^, ^6^ pancreatic cancer,^7^ breast cancer,^8^, ^9^, ^10^, ^11^, ^12^ etc., either in VMAT/IMRT or 3D radiotherapy. To manage respiratory motion, Varian's real‐time position management (RPM) system uses a reflector as a surrogate for organ motion due to patients’ respiration. An infrared camera is either mounted on the CT couch or wall/ceiling to monitor the reflector's motion. The benefit of mounting a camera on a CT couch is that the relative position of the patient and the camera is fixed during couch movement in a CT scan. Thus, the camera only detects motion of breathing from the reflector; Whereas for the wall/ceiling‐mounted camera, the breathing signal is mixed with the couch movement signal, thus a baseline drift will be added to the breathing signal during DIBH or 4D CT scanning if no measure is adopted to remove the baseline drift. The baseline drift adds extra noise to the breathing signal/waveform. It interferes with the clinical decision of gating thresholds in DIBH, and affects 4D CT reconstruction. Additionally, it will affect a patient's breathing pattern if a visual coaching device is used during CT simulation. Therefore, it may impede patient setup accuracy thus the accuracy of radiation dose delivery in treatment.^13^, ^14^, ^15^ Therefore, for the wall/ceiling‐mounted camera, a correction is needed to effectively remove the baseline drift due to couch movement in DIBH or 4D CT scanning. Varian suggests a residual baseline drift of less than 0.2 cm.^16^

**FIGURE 1:**
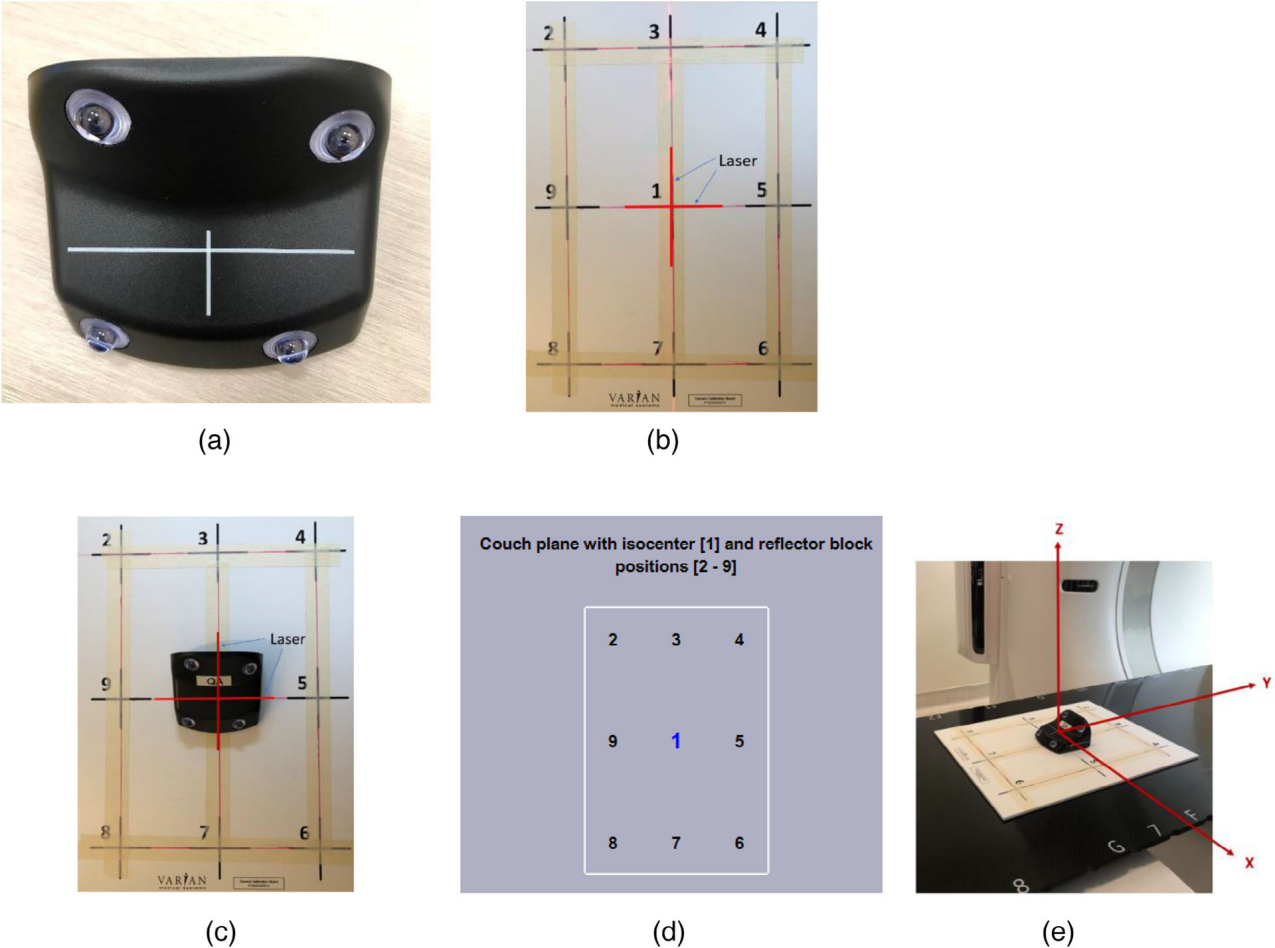
(a) Infrared reflector block; (b) Calibration plate aligned with laser; (c) Infrared reflector block placed at the isocenter and calibration plate position #1; (d) Calibration program interface for each position. (e) Couch coordinate system of CT simulation.

Respiratory Gating for Scanner (RGSC)^17^ is the successor of the RPM system, and RGSC uses the same gating principle as RPM: an infrared camera and reflective marker block with upgraded design, and both have submillimeter motion tracking accuracy.^16^ The RGSC is fully integrated with Eclipse/ARIA, and RPM is a standalone device. RGSC saves and retrieves patient waveform either in database mode or file mode, depending on RGSC system configuration. In database mode, waveform and protocol are saved in the ARIA database directly while in file mode they need to be imported into ARIA manually. In RPM, breathing waveform data is saved in a file. It needs to be converted to DICOM format, and then imported to ARIA. Unlike the marker block in RPM with six reflective markers in one plane, an RGSC marker block (Figure 1a) contains four reflective markers in three dimensions, and the RGSC tracking system uses a more sophisticated calculation algorithm resulting in finer tracking in 3D.

**FIGURE 2:**
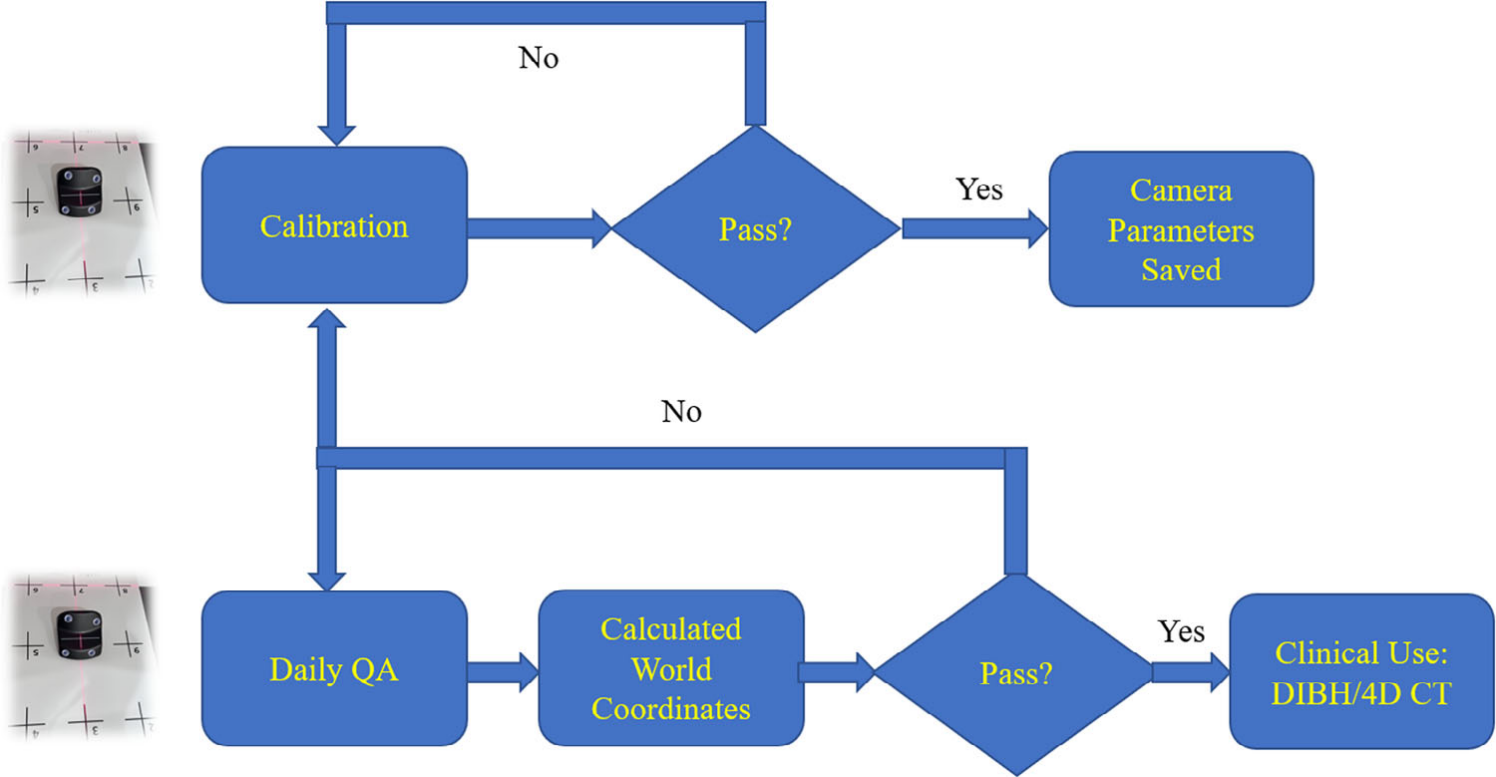
Clinical flow chart for calibration, daily QA, and DIBH/4D CT.

To remove the baseline drift introduced by couch movement during DIBH or 4D CT scanning, Varian provided a nine‐point calibration method. A plate with nine points in three rows was used to facilitate the calibration process, with a row spacing of 15 cm and column spacing of 10 cm (Figure 1b). In the calibration, a marker block was first placed on the center of the plate (Figure 1c) and aligned with the CT isocenter. Next, the calibration was started from point #1, then point #2, point #3 until point #9, following the calibration program interface (Figure 1d). After calibration, the marker block was placed back to the isocenter for verification. If the marker block is off by less than 0.5 cm in the verification of all three directions: longitudinal (Y), lateral (X), or vertical (Z) (Figure 1e), calibration is successful and camera parameters calculated from the calibrations process are saved. Otherwise, recalibration is needed. The above flow is described in the upper half of the clinic flow chart in Figure 2. On each day when there is a DIBH or 4D CT scheduled, as described in the lower half of the flow chart in Figure 2, the verification procedure above is performed as the RGSC daily QA. If the marker block is off by less than 0.5 cm in all three directions, daily QA passes, and it is ready for DIBH or 4D CT. Otherwise, recalibration is needed if daily QA fails.

The current approach for calibration uses a single marker block. With the help of lasers, one can position the marker block on positions #1, #9, and #5 accurately with negligible uncertainties, but not the other six calibration points, where the laser cannot help. During the calibration process, both the center position and the orientation of the marker block are important since the camera uses the 3D information of the four reflective markers on the marker block for tracking. The calibration uncertainty caused by the marker block positioning uncertainty on the nine calibration points will further propagate into the patient motion management. To resolve this clinical challenge, we proposed a novel calibration method using three marker blocks. In this study, we would like to compare the baseline drifts in the image acquisition of DIBH, using three reflector blocks versus using a single reflector block in the calibration of wall‐mounted RGSC camera system.

## 2 MATERIALS AND METHODS

**FIGURE 3:**
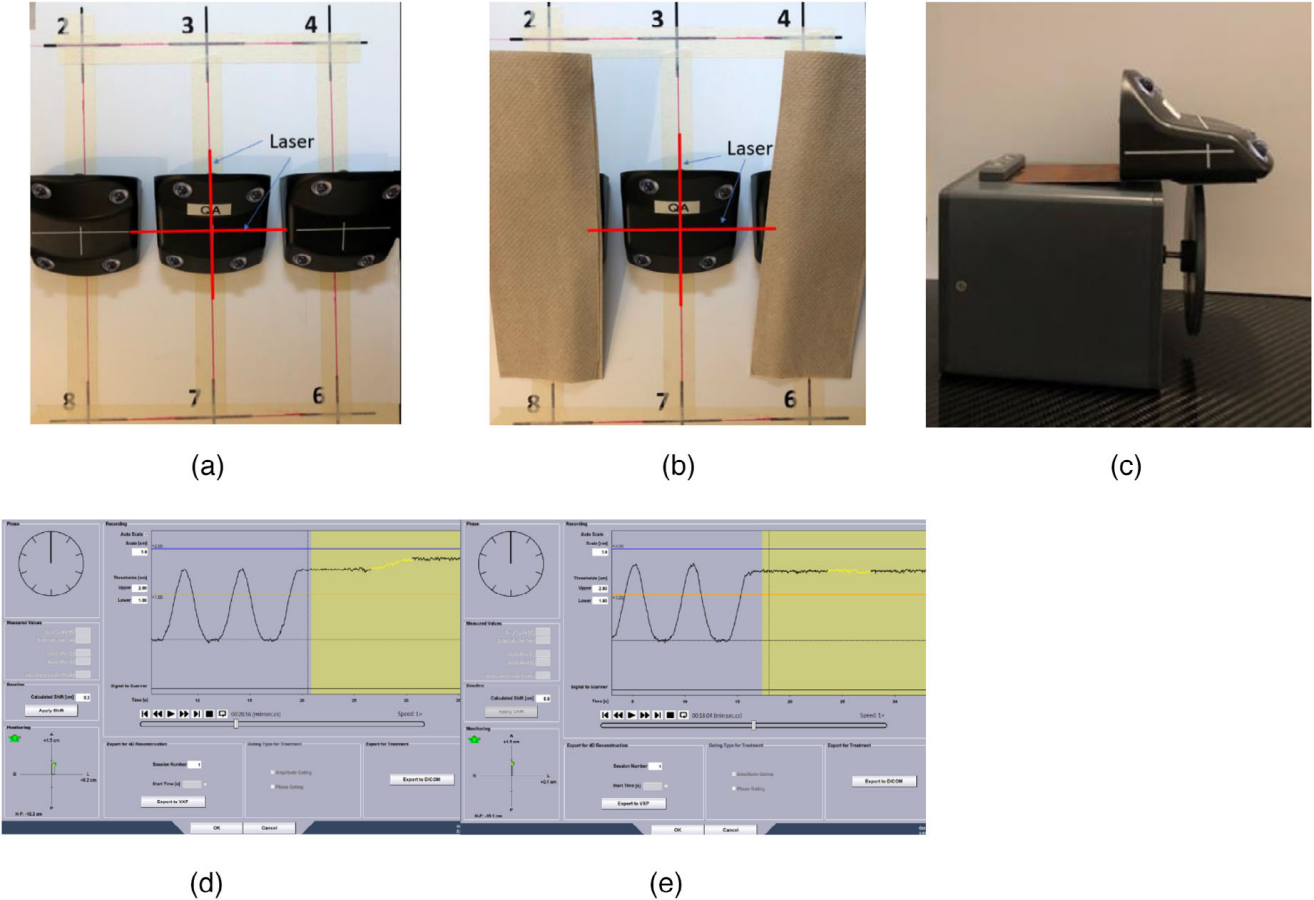
(a) Three reflector boxes aligned on positions #1, #5, and #9 under the help of laser; (b) Calibration for position #1; (c) Varian respiratory motion phantom with reflector block; (d) Baseline drifting example with single‐block calibration method for DIBH; (e) Baseline drifting example with three‐block calibration method for DIBH.

Recently, City of Hope Comprehensive Cancer Center installed two Respiratory Gating for Scanner (RGSC Ver 1.1) paired with Siemens SOMATOM go.Open Pro CT scanner. An infrared camera was mounted on the wall for each system. Instead of moving a single marker block around and repositioning it on the nine points of the calibration plate, our calibration method uses three marker blocks to remove the positioning uncertainty. Figure 3a shows the initial setup of the three‐marker block method: one marker block was placed on point #1 of the calibration plate and aligned with the isocenter, and the other two marker blocks were placed on points #5 and #9 accurately with the help of setup laser. Calibration for point #1 was performed by only exposing the marker block on point #1 to the camera while blocking the other two marker blocks on points #5 and #9 (Figure 3b). Similarly, calibration for point #5 or point #9 would be performed by blocking the other two marker blocks. Calibration of points #2, #3, and #4 was performed by moving the couch in 15 cm and exposing the calibration point only, and calibration of points #6, #7, and #8 was performed similarly by moving the couch out 15 cm. Verification after calibration was performed by moving couch back to the original position and exposing the marker block on position #1 only.

The goal of the nine‐point calibration is to effectively remove the baseline drift introduced by the relative movement between the patient and the wall‐mounted camera during DIBH/4D CT scan. To evaluate the residual baseline drift after calibration, a Varian breathing phantom with a marker block taped on it was used to simulate a patient's DIBH (Figure 3c). Using DIBH protocol, after breathing patterning learning and waiting for two breathing cycles, the breathing phantom was stopped at maximum inhale position and started DIBH CT acquisition. RGSC system recorded and saved the waveform of simulated DIBH CT scans. The yellow line segment in Figure 3d is a typical waveform segment example of the breathing phantom during couch movement in DIBH CT acquisition after single marker block calibration, where significant baseline drift was clearly present; On the other hand, Figure 3e is a typical waveform example of DIBH after three marker block calibration and it shows negligible baseline drift. For the convenience of data analysis, all baseline drifts were normalized to drifts corresponding to a typical breast CT scan length of 40 cm.

The infrared camera system imaging theory has been introduced by the American Association of Physicists in Medicine Task Group 147.^18^, ^19^ The calibration of the infrared camera will use a template placed in the physical world coordinates and reflector block to tell the camera system the location of the block. Therefore, it will build the link of the physical coordinate system with the camera view coordinate system by calculating the calibration matrix as shown in Equation (1).

(1) $$X^{\prime }\;=\;AX.$$

where X′ is a 4 × n matrix in the coordinate of the camera view and the X is a 4 × n matrix in the coordinate of the physical world view. A is a 4 × 4 calibration matrix. By using the single value decomposition, we can calculate the A matrix eigenvalue. Ideally, the eigenvalues will be 1 in the CT scanner's X, Y, and Z directions (IEC scale) so that there is no distortion of the image. However, in the real world, due to the limited view of the camera, the eigenvalues will be close to 1 with a certain uncertainty depending on the calibration procedure and operation.

The Monte Carlo simulation used random numbers to add uncertainties (± 1 mm to ± 5 mm) into the input matrix X initial location coordinates in Equation (1), the output matrix X′ was stable for the theory coordinates of the input points from the reflector boxes. The calibration matrix A in Equation (1) was then calculated each time with different uncertainties. The SVD (single value decomposition) of the calibration matrix A was performed. After the SVD decomposition, the eigenvalues could be derived, and the mean value and standard deviation were calculated. Therefore, we understand the relationship between the eigenvalues’ changes versus position uncertainties of the reflector boxes. The simulation was programmed using Matlab (Ver 9.13, The MathWorks, Inc., Natick, Massachusetts, USA)

In the physical coordinate system, it is difficult to directly quantify the magnitude of the positioning uncertainties, which include both the uncertainties of the center positions and the orientations of the marker blocks. However, we can measure the marker block position in the camera view coordinate system, which can be considered as a surrogate of the marker block position in the physical coordinate system. For example, after laser alignment of the calibration plate, we can manually place the calibration marker block on position #3 of the calibration plate and run the verification program. The verification will fail since the maker block is positioned 15 cm off from the isocenter, however, the verification will read out on the maker block's coordinates on point #3 in the camera view coordinate system. By repeating this measurement 10 times, we calculated the standard deviation of those camera view coordinates. We performed these measurements for point #3, point #7 and point #1, where only the manual positioning for point #1 used laser's help. Using this method, the uncertainties for positioning the marker block on these points were measured and compared.

## 3 RESULTS

Table 1 tabulated the sorted residual baseline drift for both methods. Although both the single marker block calibrations and three marker block calibrations passed verification, the three‐marker block calibration method showed significantly less residual baseline drift: 0.30 ± 0.12 cm for the single marker block versus 0.02 ± 0.03 cm for the three‐marker block calibration method. The difference is statistically significant with a *p*‐value less than 0.0001 using a Student *t*‐test. Considering Varian's^16^ recommendation of baseline drift less than 0.20 cm, most of the single marker block calibrations did not meet this criterion, whereas all the three‐marker block calibrations met this criterion.

**TABLE 1:** Comparison of the residual drifts of simulated DIBH waveforms for single marker block calibration versus three‐marker block calibration.

Single marker block (cm)	Three‐marker blocks (cm)
0.15	0.0
0.21	0.02
0.22	0.02
0.27	−0.02
0.27	0.03
0.28	0.03
0.29	0.03
0.33	−0.03
0.34	0.07
0.60	0.08
0.30 ± 0.12	0.02 ± 0.03

The DIBH scan was simulated using Varian's respiratory motion phantom (Figure 3c).

**FIGURE 4:**
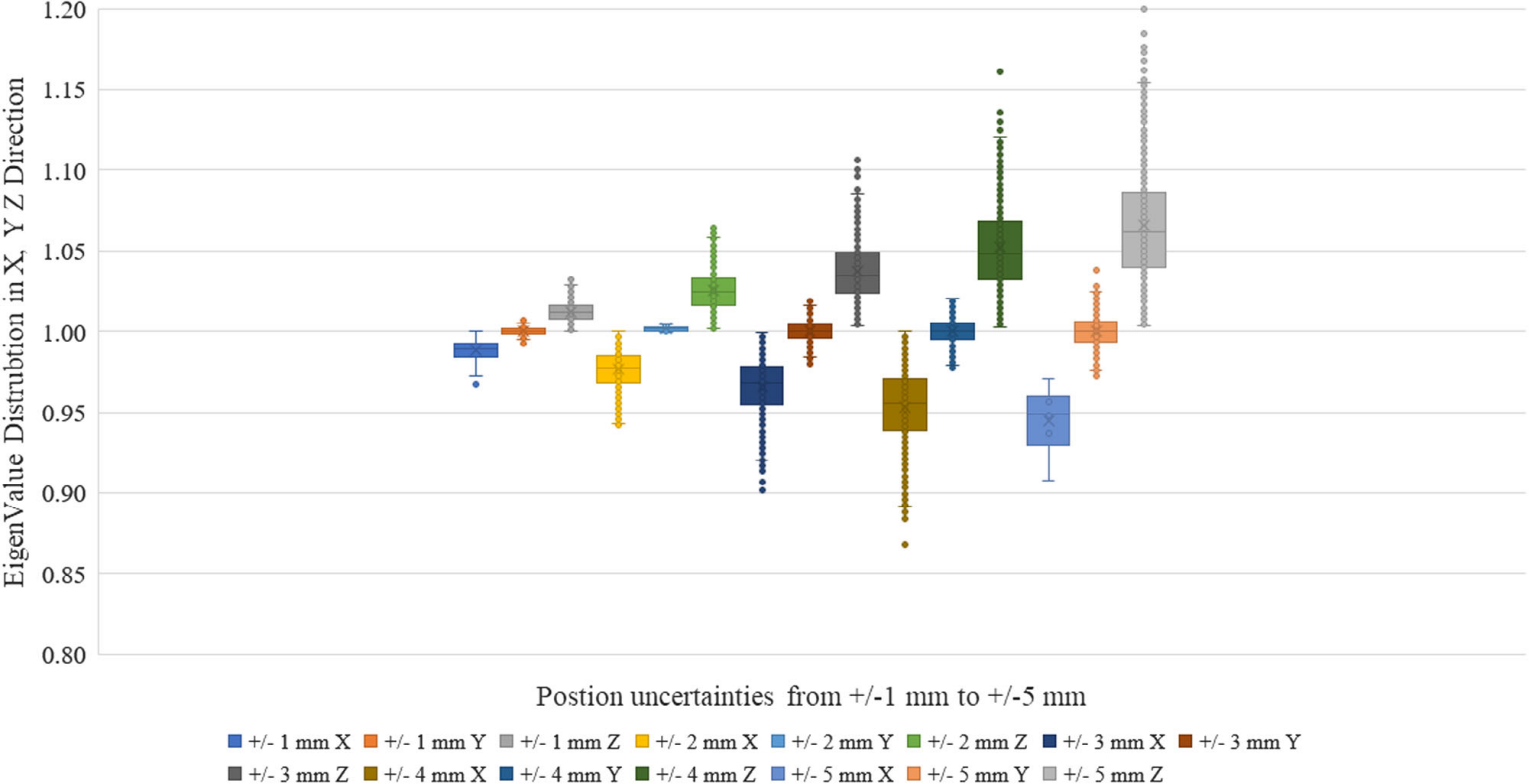
Box& Whisker plot of different position uncertainties in X, Y, and Z direction of the reflector boxes versus the calibration matrix of the camera eigenvalue changes. The results were based on Monte Carlo simulation with different uncertainties added into the calibration reflector boxes.

Figure 4 shows the Monte Carlo simulation results with position uncertainties ± 1  to ± 5 mm. With position uncertainty increasing from ± 1  to ± 5 mm, the eigenvalues in the X, Y, and Z directions will also change with a larger distribution range. The current design template for calibration is not sensitive in the Y direction. However, it will be sensitive to X and Z directions. To be within 0.5 mm tolerance, the position uncertainties could be up to ± 3 mm during the calibration process based on the Monte Carlo simulation results.

Table 2 compared the positioning uncertainties on point #1, point #3, and point #7 between the manual positioning and the positioning using couch move. One can see that the uncertainties using couch move are dramatically lower in the longitudinal and lateral directions than those using manual positioning for all points. The uncertainties of the vertical direction are negligible since the calibration plate is leveled so the marker block will have almost the same vertical coordinate anywhere on the plate. Also, one can see that, for manual positioning, the uncertainties of the marker block on point #1 are much less than those on point #3 and point #7, because positioning the marker block on point #1 used laser's help while positioning the marker block on point #3 and #7 could not use the laser's help. Most importantly, the uncertainties for manual positioning marker block on point #3 and point #7 are close or larger than 0.15 cm in the longitudinal and lateral directions, while the uncertainties on all the points are negligible in all directions for positioning the marker block using couch move. The uncertainties of the coordinate difference between points #3 and #7 are also shown in the last row of Table 2. Assuming the coordinate uncertainty follows a normal distribution, 32% or 5% of the values will fall beyond 1 or 2 standard deviations (SD) of the expectation values. Excluding points #1, #5, and #9 due to their negligible positioning uncertainty under laser help, there are 6C2 = 15 pairs of combination among points #2, #3, #4, #6, #7, and #8. It is almost certain that the coordinate differences for some pairs of points are larger than 1 SD in either the longitudinal direction or the lateral direction or both; and it is highly likely that some coordinate differences are larger than 2 SD, which is more than 0.4 cm.

**TABLE 2:** Coordinate uncertainty comparison in camera view coordinate system, between manual positioning and positioning using couch move.

SD (standard deviation)	Vertical (cm)	Longitudinal (cm)	Lateral (cm)
Point #1 (manual with laser)	0.020	0.041	0.041
Point #1 (couch move)	0.006	0.016	0.000
Point #3 (manual)	0.020	0.173	0.141
Point #3 (couch move)	0.005	0.025	0.000
Point # 7 (manual)	0.024	0.165	0.157
Point #7 (couch move)	0.008	0.025	0.000
(Point #3 – Point #7) (manual)	0.031	0.239	0.211

## 4 DISCUSSION

The purpose of the RGSC calibration is to remove the baseline drift introduced by couch movement during the DIBH or 4D CT scanning process. The RGSC calibration process calculates the transformation matrix from the physical coordinate system to the camera view coordinate system, and the verification process verifies the isocenter in the physical coordinate system is the isocenter in the camera view system. A Varian breathing phantom was used to validate the effectiveness of baseline removal after calibration. The significant residual baseline drift after Varian's one‐block calibration method indicates this calibration method needs improvement. Monte Carlo simulation shows the importance of positioning accuracy in the calibration process by analyzing the error propagation, and the marker block position measurement in the camera view coordinate system shows that the marker block positioning uncertainty in Varian's single block calibration method is not negligible.

In the three‐marker block calibration method, the initial positioning of the three marker blocks can be accurately performed with the help of the CT laser, and positioning the marker blocks to other positions can be performed by moving the couch, which has a submillimeter accuracy, thus the marker block positioning uncertainty is minimized. It is important to make sure the couch offset is within tolerance so that the systematic bias is negligible. The key difference between this method and the single marker block method is that this method uses couch move, instead of manually positioning the marker block on the calibration plate as in the single marker block method. Therefore, according to Table 2, the single marker block method introduces significant positioning uncertainty, which is the cause of the residual baseline drift in DIBH; whereas the baseline drift was effectively removed using the three‐marker block calibration method as positioning uncertainty is negligible. To further facilitate the calibration, the three marker blocks can be glued to positions #1, #5, and #9 on the calibration plate thus one can align all three maker blocks with the laser at the same time. We perform this calibration every month and perform the calibration as needed when we notice there is significant baseline drift either in the DIBH or 4DCT scan. Verification is performed each day when we have a DIBH or 4DCT scan. It is important to use the center block of the three marker blocks for verification, considering the manufacturing variation of marker blocks.

The single marker block calibration method proposed by Varian introduces significant baseline drift. As seen in Table 1, one can see that only one out of ten single marker block calibrations meet the baseline drift criterion recommended by Varian.^16^ Even if one considers the baseline drifts of 0.21  and 0.22 cm as roughly meeting the criterion, still only 30% meet. That means, on average, one needs to perform single block calibration three to four times to meet the criterion recommended by Varian. Whereas the three‐marker block calibration meets the criterion every time, thus saving calibration time dramatically.

Using the same idea as the three‐marker block calibration method, a much more time‐consuming variant method using a single marker block can also be used to minimize the marker block position uncertainty if one cannot find three marker blocks for calibration. In this single marker block variant method, one can accurately place the single marker block on positions #1, #5 and #9, as is done in the three‐marker block method using laser's help. To place the single marker block at each of the positions #2, #3, #4 and #6, #7, #8, we move the couch so that the center line of the calibration plate and the single marker block are aligned with CT laser, then move the couch accordingly to these positions, instead of manually placing the marker block to these positions. Using this method, similar results can be achieved as the three‐marker block method. However, this method increases the calibration time dramatically: it involves 7 laser alignments and 12 couch movements following the Varian calibration procedure from point #1 to point #9; while the three‐marker block method needs only 1 laser alignment and 4 couch movements. If Varian's calibration algorithm can be modified so that one can calibrate in the order of points #1, #3, #7, then points #9, #2, #8 followed by points #5, #4, and #6, the single marker block variant calibration method can be performed with three laser alignments and six couch movements.

The major theory behind the camera calibration is the camera coordinates X′ will use the calibration matrix A and input coordinates X. X′ = AX or A = X′X−1. When the calibration coordinates X has fewer uncertainties, the calibration matrix A will be more accurate, thus the camera coordinates X′ will have fewer uncertainties. As in the three‐marker block calibration method, we use the laser and couch move to place the calibration marker block on all the calibration positions from position #1 to #9, thus human uncertainty is significantly reduced.

## 5 CONCLUSION

We proposed a three‐marker block method for the wall mount RGSC camera calibration. This method minimizes the marker block positioning uncertainty and effectively removes the baseline drift caused by couch movement in DIBH and 4D CT simulation.

## AUTHOR CONTRIBUTIONS

All authors made substantial contributions to the design of the work, data analysis and interpretation, and preparation of the manuscript.

## CONFLICT OF INTEREST STATEMENT

This research has no conflict of interest.
